# Roles of prostaglandin F2alpha and hydrogen peroxide in the regulation of Copper/Zinc superoxide dismutase in bovine corpus luteum and luteal endothelial cells

**DOI:** 10.1186/1477-7827-10-87

**Published:** 2012-10-26

**Authors:** Hai V Vu, Tomas J Acosta, Shin Yoshioka, Hironori Abe, Kiyoshi Okuda

**Affiliations:** 1 Laboratory of Reproductive Physiology, Graduate School of Natural Science and Technology, Okayama University, Okayama, 700-8530, Japan

**Keywords:** Bovine, Corpus luteum, Prostaglandin F_2α_, Hydrogen peroxide, Superoxide dismutase, Luteal endothelial cell

## Abstract

**Background:**

Prostaglandin F2alpha (PGF) induces luteolysis in cow by inducing a rapid reduction in progesterone production (functional luteolysis) followed by tissue degeneration (structural luteolysis). However the mechanisms of action of PGF remain unclear. Reactive oxygen species (ROS) play important roles in regulating the luteolytic action of PGF. The local concentration of ROS is controlled by superoxide dismutase (SOD), the main enzyme involved in the control of intraluteal ROS. Thus SOD seems to be involved in luteolysis process induced by PGF in cow.

**Methods:**

To determine the dynamic relationship between PGF and ROS in bovine corpus luteum (CL) during luteolysis, we determined the time-dependent change of Copper/Zinc SOD (SOD1) in CL tissues after PGF treatment in vivo*.* We also investigated whether PGF and hydrogen peroxide (H2O2) modulates SOD1 expression and SOD activity in cultured bovine luteal endothelial cells (LECs) in vitro.

**Results:**

Following administration of a luteolytic dose of PGF analogue (0 h) to cows at the mid-luteal stage, the expression of *SOD1* mRNA and protein, and total SOD activity in CL tissues increased between 0.5 and 2 h, but fell below the initial (0 h) level at 24 h post-treatment. In cultured LECs, the expression of *SOD1* mRNA was stimulated by PGF (1–10 microM) and H2O2 (10–100 microM) at 2 h (P<0.05). PGF and H2O2 increased SOD1 protein expression and total SOD activity at 2 h (P<0.05), whereas PGF and H2O2 inhibited SOD1 protein expressions and total SOD activity at 24 h (P<0.05). In addition, H2O2 stimulated PGF biosynthesis at 2 and 24 h in bovine LECs. Overall results indicate that, SOD is regulated by PGF and ROS in bovine LECs. SOD may play a role in controlling intraluteal PGF and ROS action during functional and structural luteolysis in cows.

## Background

Prostaglandin F_2α_ (PGF) from the uterus or from the ovary is responsible for the regression of the corpus luteum (CL) in mammals [1,2]. In vivo studies in cows demonstrated that intramuscular injections of PGF analogues given in the mid-luteal stage induce an acute decrease in progesterone (P_4_) production (functional luteolysis) followed by tissue degradation and a decrease in size of the CL (structural luteolysis) [2]. On the other hand, in vitro studies showed that direct treatment of pure populations of luteal steroidogenic cells (LSCs) with PGF does not inhibit basal P_4_ production by the large LSCs, and stimulates P_4_ production by the small LSCs and by a mixture of large and small LSCs [3,4] suggesting that PGF action differs in each type of luteal cells. The exact mechanism involved in the luteolytic cascade initiated by PGF remains unclear. Interestingly, several studies reported the presence of PGF receptors in luteal endothelial cells (LECs) and that LECs respond to PGF stimulation [5,6], suggesting that LECs are a target for PGF, and that LECs play roles in the local mechanism of CL regression induced by PGF.

Reactive oxygen species (ROS) including hydrogen peroxide (H_2_O_2_), superoxide anion (O_2_^-^) and hydroxyl radical (OH^-^) have been implicated in the luteolytic process [7]. In rats, PGF induces a decrease in serum concentrations of P_4_ in association with increasing generation of superoxide anion and H_2_O_2_ in the luteal tissue [8]. Treatment of LSCs with PGF induces ROS production and apoptosis [9]. Moreover, H_2_O_2_ was found to stimulate PGF production in human endometrial stromal cells [10]. We recently observed that an injection of PGF induces a transient (1–2 h) increase in the partial pressure of oxygen (pO_2_) in ovarian venous blood [11], and that the pO_2_ of venous blood is higher in the ovarian vein than in the jugular vein in cow. Therefore, the luteal microenvironment seems to be exposed to high O_2_ condition, especially during the short period of time (1–2 h) following PGF treatment.

Superoxide dismutase (SOD) acts as an antioxidant enzyme in nearly all cells exposed to oxygen. SOD catalyzes the dismutation of superoxide into oxygen and H_2_O_2_. Mammalian cells have three forms of SOD. Copper/Zinc (SOD1) is present in the cytosol, nucleus and the inter-membrane space of mitochondria; Manganese SOD (SOD2) is a manganese-containing enzyme that is present in the mitochondrial matrix; Extracellular SOD (SOD3) is a secreted copper-containing protein that is found in the extracellular matrix of tissues [12]. SOD1 dismutates superoxide radicals resulting from cellular oxidative metabolism into H_2_O_2_[13] represents 80% of the total SOD found in the rat CL [14]. In addition, a decrease in intracellular SOD activity was accompanied by a decrease of P_4_ production by rat CL [15]. However, the local mechanisms controlling the luteolytic actions of PGF in LECs remain unknown. We hypothesized that SOD, the main enzyme involved in the control of intraluteal ROS, is differently regulated at the time of functional (2 h) and structural (24 h) luteolysis.

To test the above hypothesis, we determined the time-dependent changes of SOD in CL tissues after PGF treatment in vivo and the influence of PGF and H2O2 on SOD1 expression and SOD activity in bovine LECs in vitro.

## Methods

### Collection of CL tissue during PGF-induced luteolysis

The animal procedures were approved by the local institutional animal care and use committee of Polish Academy of Sciences in Olsztyn, Poland (Agreement No. 5/2007, 6/2007 and 88/2007). Healthy normally cycling Polish Holstein Black and White cows were used for collection of CL. Estrus was synchronized in the cows by two injections of an analogue PGF (Dinoprost, Dinolytic; Pharmacia & Upjohn, Belgium) with an 11-days interval. Ovulation was determined by a veterinarian via transrectal ultrasonography examination. Then, CLs were collected by Colpotomy technique using a Hauptner’s effeninator (Hauptner and Herberholz, Solingen, Germany) on day 10 post-ovulation, i.e., just before administration of a luteolytic dose of analogue PGF (Dinoprost, Dinolytic; Pharmacia & Upjohn, Belgium) (0 h), and at 0.5, 2, 12 and 24 h post-treatment (n=4 per time-point). CL tissues were dissected from the ovary and then stored at −80°C until the *SOD1* mRNA, protein and total SOD activity analysis.

### Bovine LEC isolation and cell culture

LECs were isolated from five CLs at the mid-luteal phase (days 8–12 of the estrous cycle) [16] using magnetic beads as previously described [17] and recently validated in our laboratory [6,18]. Briefly, magnetic tosylactivated beads (Dynabeads M-450, 140.04; Dynal ASA, Oslo, Norway) were coated with 0.15 mg/ml lectin from *Bandeiraea simplicifolia* (BS-1; L2380; Sigma-Aldrich, St. Louis, MO, USA), which specifically binds the glycoproteins expressed by bovine LECs [17]. Luteal cell (LC) suspension was mixed with beads at a concentration of 4 × 10^8^ beads/ml, and incubated for 20 min at 4°C on a rocking platform. The cells were pooled and cultured in endothelial cell (EC) growth medium (MV 2; C22121; Promo Cell, Heidelberg, Germany) at 37°C in a humidified atmosphere of 5% CO_2_ in air. Only colonies with a homogeneous cell population were removed with a pipette and cultured in collagen-coated 25 cm^2^ culture flasks (690175; Greiner Bio-One, Frickenhausen, Germany). The cultures and passages were repeated until a homogeneous population of pure LECs was obtained. The LECs used in the present study were previously confirmed to be positively stained with rabbit anti-human von Willebrand factor (vWF, F3520; Sigma-Aldrich), isolated LECs expresses CD31 but not *3β*-hydroxysteroid dehydrogenase (*3β-HSD*) mRNA as reported previously [6,17,18]. Experiments were performed on confluent cultures and the cells (LECs) were from passage 2–5.

The LECs were seeded at a concentration of 1 × 10^5^ viable cells/ml into 24-well plates (662160; Greiner Bio-One) for determination of *SOD1* mRNA or PGF production, and 1 × 10^6^ viable cells/ml into 75-cm^2^ culture flasks (658175; Greiner Bio-One) for determination of SOD1 protein expression and total SOD activity in culture cells. LECs were cultured in DMEM/F-12 (D/F; D8900; Sigma-Aldrich) supplemented with 10% calf serum (v/v, 16170–078; Life Technologies Inc., Grand Island, NY, USA), 20 μM gentamicin (G1397; Sigma-Aldrich) and 2 μM amphotericin B (A9528; Sigma-Aldrich). The cells reached confluence on 5^th^ day of culture. After the cells reached confluence, the medium was replaced with fresh D/F supplemented with 5 μg/ml holo-transferrin (T3400; Sigma-Aldrich), 500 μM ascorbic acid (013–12061; Wako Pure Chemical Industries, Ltd., Osaka, Japan), 5 nM sodium selenite (S5261; Sigma-Aldrich), and 0.1% (w/v) BSA (10 735 078 001; Roche Diagnostics, Mannheim, Germany).

### Cell viability test

LECs cultured in 96-well plates were exposed to H_2_O_2_ (0.1 – 1000 μM) for the final 24 h of culture. The cell viability was determined by Dojindo Cell Counting Kit including WST-1 (Dojindo, Kumamoto, Japan; No. 345–06463). Briefly, WST-1, a kind of MTT [3-(4,5-dimethyl-2 thiazolyl)-2,5-diphenyl-2 H-tetrazolium bromide], is a yellow tetrazolium salt that is reduced to formazan by live cells containing active mitochondria. The culture medium was replaced with 100 μl D/F without phenol red medium-BSA, and a 10-μl aliquot (0.3% WST-1, 0.2 mM 1-methoxy PMS in PBS, pH 7.4) was added to each well. The cells were then incubated for 4 h at 38°C. The absorbance (A) was read at 450 nm using a microplate reader (Bio-Rad, Hercules, CA; Model 450). Percentage of cytotoxicity was determined by subtracting the mean A of H_2_O_2_-treated wells (A_test_) from the mean A of untreated wells (A_control_) and then dividing by the mean A of untreated wells (A_control_). The mean A of wells in the absence of the cells was subtracted from the mean A of all experimental wells. The percent cytotoxicity was calculated as 100 × (A_control_ – A_test_)/(A_control_).

### Dose-dependent effect of PGF and H_2_O_2_ on *SOD1* mRNA expression at 2 h in vitro

To determine whether LECs were responsive to increased concentrations of PGF and H_2_O_2_ in the present culture system, LECs cultured in 24-well plates were incubated for 2 h with or without 0.1-10 μM PGF or 1–100 μM H_2_O_2_ (n=4 experiments). The cells were then collected and stored at −80° C for the analysis of *SOD1* mRNA expression.

### SOD1 protein and total SOD activity at 2 h and at 24 h in vitro

To examine whether SOD is differently regulated by PGF and H_2_O_2_ at the time of functional and structural luteolysis, LECs cultured in 75-cm^2^ culture flasks were incubated for 2 h (mimicking functional luteolysis) and 24 h (mimicking structural luteolysis) with or without PGF (1 μM) or H_2_O_2_ (10 μM). The samples were then used for analysis of SOD1 protein expression and total SOD activity (n=4 experiments). The concentrations of PGF and H_2_O_2_ were chosen based on the result of *SOD1* mRNA expression in vitro, and the effect of H_2_O_2_ on cell viability

### PGF production in vitro

To determine the dose-dependent effects of H_2_O_2_ on PGF production, LECs cultured in 24-well plates were exposed to H_2_O_2_ (1–100 μM) for 2 h or 24 h. After incubation, the conditioned media were collected in 1.5 ml tubes containing 5 μl of a stabilizer solution (0.3 M EDTA, 1% (w/v) acid acetyl salicylic, pH 7.3) and frozen at −30°C until the PGF assay (n=4 experiments).

### Reverse transcript-PCR

Total RNA was prepared from the CL tissue or cultured bovine LECs using TRI reagent according to the manufacturer’s direction (TRI Reagent RNA isolation protocol, © 2008 Ambion, Inc). One microgram of total RNA of each sample was reverse transcribed using a SuperScript First-Strand Synthesis System for RT-PCR (11904–018; Invitrogen), and the reaction mixture was used in each PCR together with the appropriate oligonucleotide primer pairs. The primer for SOD and beta-actin (ACTB) were designed and characterized as described previously [19]. Primer for SOD1 was: forward: AAGGCCGTCTGCGTGCTGAA; reverse: CAGGTCTCCAACATGCCTCT (accession No: M81129; product: 240 bp). Primer for ACTB was: Forward: CGGCATTCACGAAACTACC; Reverse: ATCAAGTCCTCGGCCACAC (accession No: AY141970; product: 536).

The RT-PCRs were conducted with the house-keeping gene ACTB as an internal standard. ACTB primer was added at the appropriate cycle number by the “primmer-dropping method” as described by Wong et al. [20] with modification [21]. The PCRs were carried out using TaKaRa Taq (R001A; Takara Bio Inc., Shiga, Japan) and a thermal cycler (iCycler; Bio-rad Laboratories, Hercules, CA, USA). The PCR conditions were as follow: activation of DNA polymerase for 20 sec at 95°C, annealing for 1 min at 60°C, and extension for 1 min at 70°C, follow by final extension for 5 min at 72°C. The number of cycles was 27 for SOD and 23 for ACTB. Two-fifths aliquot of each reaction mixture was electrophoresed on a 1.5% agarose gel containing ethidium bromide with a known standard (100-bp ladder, New England Biolabs Inc., Beverely, MA, USA; #N3231S) and photographed under ultraviolet illumination. The integrated density was determined by ImageJ software (Windows version of NIH Image, http://rsb.info.nih.gov/nih-image/, National Institutes of Health). Relative density was quantified by normalization of the integrated density of each corresponding β-actin.

### SOD1 protein expression

SOD1 protein expression in luteal tissue and in cultured bovine LECs was assessed by Western immunoblotting analysis. Tissues or cells were lysed in 150 μl lysis buffer (20 mM Tris–HCl, 150 mM NaCl, 1% Triton X-100 [Bio-Rad Laboratories], 10% glycerol [G7757; Sigma-Aldrich], Complete [11 697 498 001; Roche Diagnostics, Basel, Switzerland], pH 7.4). Protein concentrations in the lysates were determined by the method of Osnes et al. [22], using BSA as a standard. The proteins were then solubilized in SDS gel-loading buffer (10% glycerol, 1% β-mercaptoethanol [137–06862; Wako Pure Chemical Industries, Ltd.], pH 6.8) and heated at 95°C for 10 min. Samples (50 μg protein) were electrophoresed on a 15% SDS-PAGE for 1.5 h at 30 mA. The separated proteins were electrophoretically transblotted to a 0.2-μM nitrocellulose membrane (LC2000; Invitrogen) at 300 mA V for 3 h in transfer buffer (25 mM Tris–HCl, 192 mM glycine, 20% methanol, pH 8.3). The membrane was washed in TBS-T (0.1% Tween 20 in TBS [25 mM Tris–HCl, 137 mM NaCl, pH 7.5]), incubated in blocking buffer (5% nonfat dry milk in TBS-T) for 1 h at room temperature, incubated at 4°C with a primary antibody specific to each protein (goat SOD1 polyclonal antibody [23 kDa; 1:500 in TBS-T, overnight; sc-8637; Santa Cruz Biotechnology, Santa Cruz, CA, USA] and mouse ACTB monoclonal antibody [internal standard, 42 kDa; 1:4000 in TBS-T, overnight; A2228; Sigma-Aldrich]). The membrane was washed three times for 5 min in TBS-T at room temperature, incubated with secondary antibody (SOD1 [1:10000 in TBS-T]: anti-goat Ig, HRP-linked whole antibody produced in donkey, sc-2020; Santa Cruz; ACTB [1:40000 in TBS-T]: anti-mouse Ig, HRP-linked whole antibody produced in sheep, NA931; Amersham Biosciences, Buckinghamshire, UK) for 1 h, and washed three times in TBS for 5 min at room temperature. The signal was detected by a ECL Western immunoblotting detection system (RPN2109; Amersham Biosciences). The intensity of the immunological reaction was estimated by measuring the optical density in the defined area by computerized densitometry using NIH Image (National Institutes of Health; Bethesda, MD, USA).

### Total SOD activity assay

Total SOD activity was determined in CL tissues collected at 0, 0.5, 2, 12, and 24 h after PGF injection and in the LECs at the end of a 2-h or 24-h incubation period using SOD Assay Kit-WST (S311-08; Dojindo Laboratories, Kumamoto, Japan). Total SOD activity was calculated using a concurrently run SOD standard curve, and expressed as inhibition rate or percentage of control (raw data on total SOD activity was normalized based on protein concentrations, units per mg of cellular protein).

### Determination of PGF concentration

The concentration of PGF in the culture medium was determined by enzyme immunoassay (EIA) as described previously [18]. The PGF standard curve ranged from 15.625 to 4000 pg/ml, and the median effective dose (ED_50_) of the assay was 250 pg/ml. The intra- and inter-assay coefficients of variation were 7.4 and 11.6%, respectively. The cross-reactivities of the antibody were 100% for PGF, 3.0% for PGD2, 1.1% for PGI, 0.15% for PGE2, and < 0.03% for PGA2. The DNA content, estimated using the spectrophotometric method by Labarca & Paigen [23], was used to standardize the PGF concentrations.

### Statistical analysis

Data of *SOD1* mRNA, protein level and total SOD activity were obtained in four separate experiments. PGF concentration and cell viability were performed in triplicate samples for each experimental group of LECs or CL tissues (in vivo). The statistical significances of differences in the amounts of *SOD1* mRNA, protein levels, total SOD activity, cell viability and differences in PGF concentrations were analyzed using two-way analysis of variance (ANOVA) with repeated-measures or one-way ANOVA followed by Fisher’s protected least-significant difference (PLSD) procedure as multiple comparison tests. All values were expressed as the mean ± SEM of four separate experiments. A level of *P*<0.05 was considered to be statistically significant.

## Results

### *SOD1* mRNA, protein expression and total SOD activity during PGF-induced luteolysis in vivo

**Figure 1:**
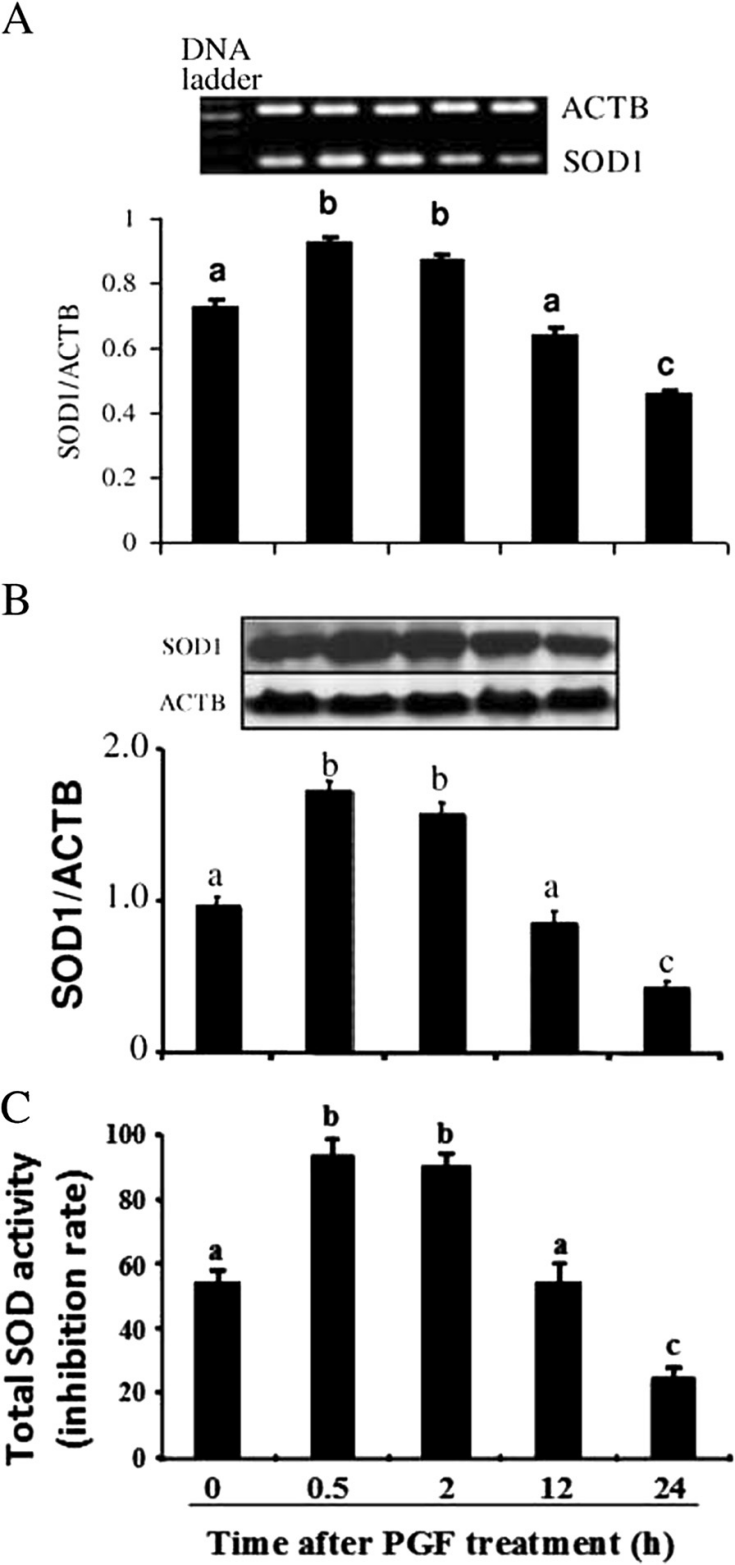
**Change of SOD1 in bovine CL tissue after PGF injection.** Effect of a PGF analogue administration on the expression of *SOD1* mRNA (**Figure**1A), protein (**Figure**1B) expression and total SOD activity (**Figure**1C) in CL tissues on Day 10 of the estrous cycle (n=4 cows/group). Different subscript letters indicate significant differences (P<0.05) between the PGF-treated (0.5, 2, 12, and 24 h) and control groups (0 h).

Injection of a luteolytic dose of PGF increased the expression of *SOD1* mRNA (Figure 1A), *SOD1* protein (Figure 1B) and total SOD activity (Figure 1C) from 0.5 to 2 h in bovine CL tissues, but decreased at 24 h (P<0.05).

### Dose-dependent effects of H_2_O_2_ on LECs viability

**Figure 2:**
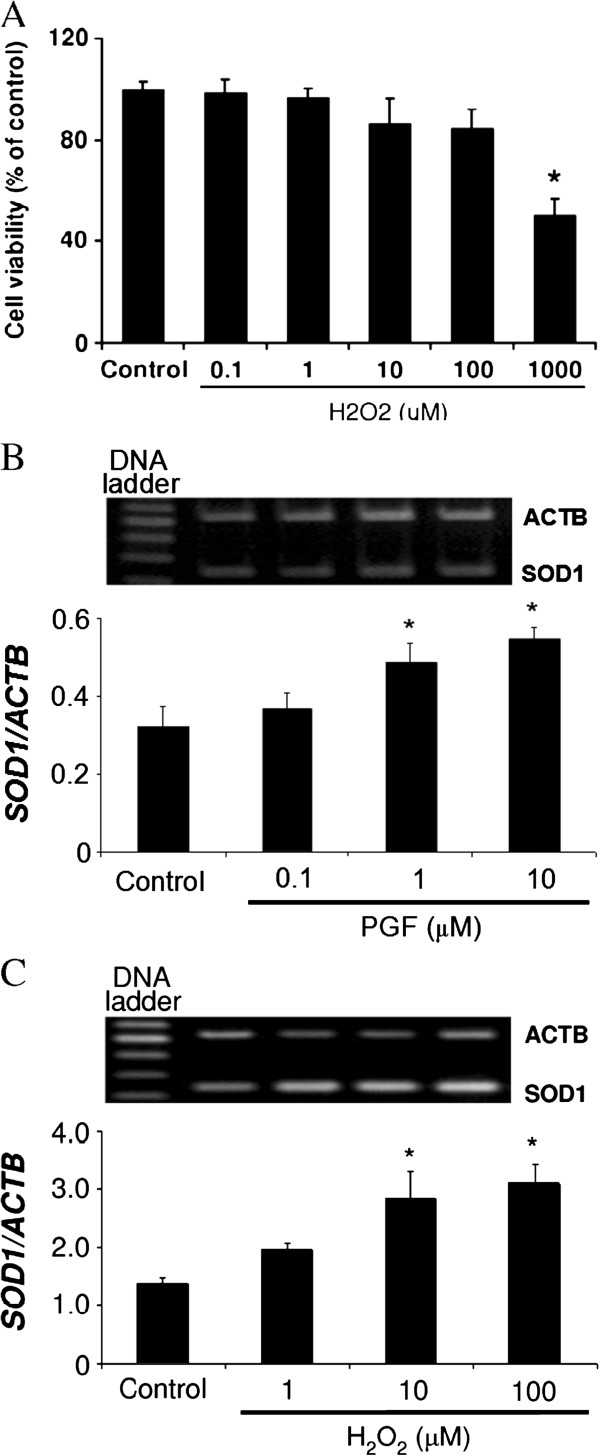
**Cell viability test and *SOD1 *mRNA expression in LECs.** Dose-dependent effects of H_2_O_2_ on the cell viability and dose-dependent effects of PGF and H_2_O_2_ on *SOD1* mRNA expression in cultured bovine LECs. The cells were cultured with H_2_O_2_ (1, 10, 100 and 1000 μM) (**Figure**2A) for 24 h for testing cell viability and with PGF (0.1, 1 and 10 μM) (**Figure**2B) and H_2_O_2_ (1–100 μM) (**Figure**2C) for 2 h to evaluate of *SOD1* mRNA expression change. Photographs showed the expression of *SOD1* (240 bp) and *ACTB* (536 bp) mRNA. The amount of *SOD1* mRNA is expressed relative to the amount of *ACTB* mRNA. Asterisks indicate significant differences between treated and untreated cells (P<0.05). All values represent mean ± SEM of four separate experiments.

H_2_O_2_ at the concentrations from 1–100 μM did not cause significantly cell dead compared with control. However, H_2_O_2_ at 1000 μM reduced significantly viability of LECs (Figure 2A).

### Dose-dependent effect of PGF and H_2_O_2_ on *SOD1* mRNA expression at 2 h in vitro

Both PGF (1-10 μM; Figure 2B) and H_2_O_2_ (10–100 μM; Figure 2C) induced mRNA expression of SOD1 in cultured bovine LECs incubated for 2 h (P<0.05). However the lower doses of PGF (0.1 μM) and H_2_O_2_ (1 μM) did not affect significantly *SOD1* mRNA expression.

### SOD1 protein and total SOD activity at 2 h and at 24 h in vitro

**Figure 3:**
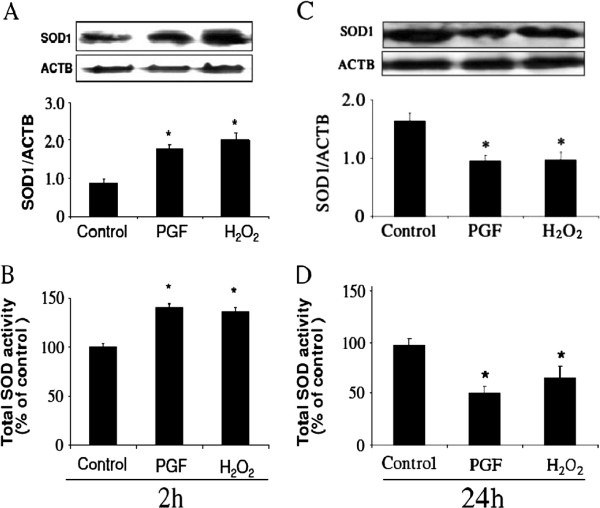
**SOD1 protein expression and total SOD activity in LECs.** Effects of PGF and H_2_O_2_ on the expression of SOD1 protein (**Figure**3A, 3C) and total SOD activity (**Figure**3B, 3D) in cultured bovine LECs. The cells were cultured with 1 μM PGF and 10 μM H_2_O_2_ for 2 and 24 h. Photographs showed the expression of SOD1 protein (23 kDa) and ACTB (42 kDa). The amount of SOD1 protein is expressed relative to the amount of ACTB. Total SOD activity was determined by a colorimetric method using SOD Assay kit-WST. Asterisks indicate significant differences between untreated cells and treated cells (P<0.05). All values represent mean ± SEM of four separate experiments.

PGF and H_2_O_2_ significantly stimulated SOD1 protein expression (Figure 3A) and total SOD activity (Figure 3B) at 2 h (P<0.05) in cultured LECs. However, PGF and H_2_O_2_ decreased SOD1 protein expression (Figure 3C) and total SOD activity (Figure 3D) at 24 h (P<0.05) in cultured LECs.

### PGF production in vitro

**Figure 4:**
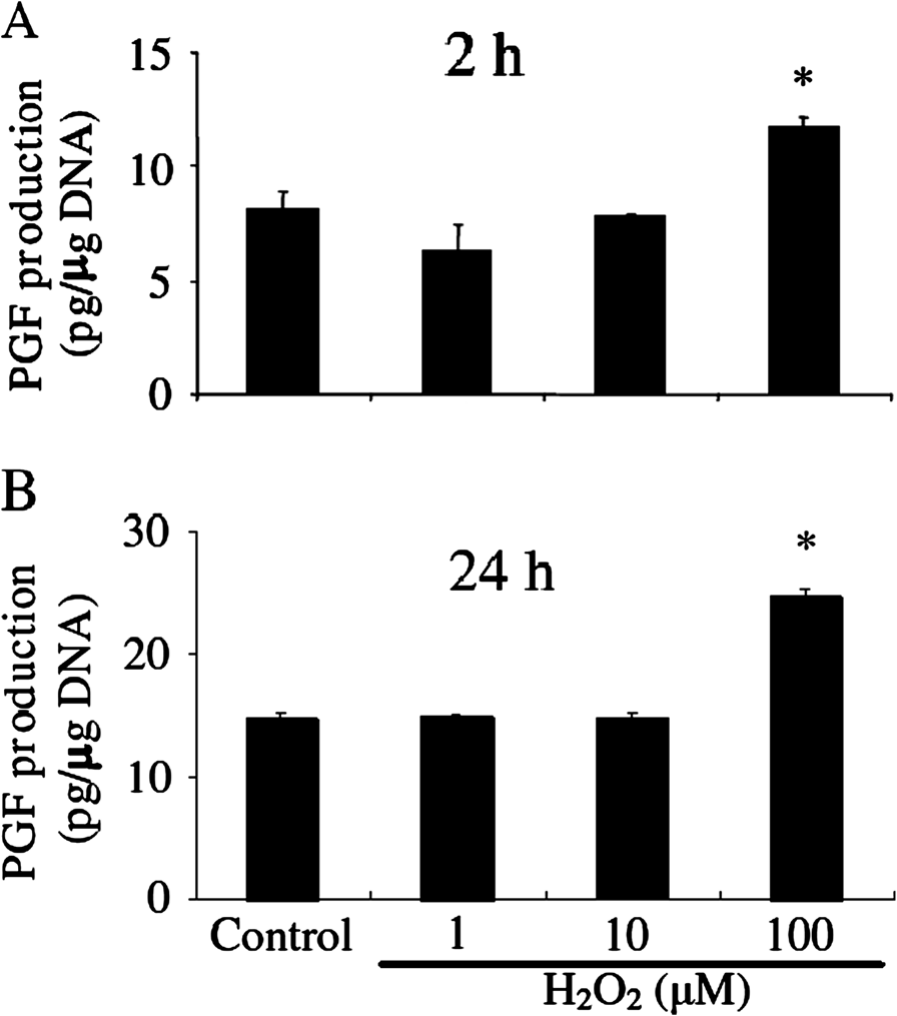
**Effect of H**_**2**_**O**_**2**_** on PGF production in LECs.** Dose-dependent effects of H_2_O_2_ on PGF production in cultured bovine LECs. The cells were cultured with H_2_O_2_ (1, 10 and 100 μM) for 2 h (**Figure**4A) and 24 h (**Figure**4B). All values represent mean ± SEM of four separate experiments. Asterisks indicate significant differences compared with untreated cells (P<0.05).

In cultured LECs, PGF production was simulated by 100 μM H_2_O_2_, but not by lower concentrations at 2 h (Figure 4A) and 24 h (Figure 4B) (P<0.05).

## Discussion

The present study demonstrated that administration of a luteolytic dose of PGF increased the expression of SOD1 in the CL tissue between 0.5 and 2 h post-treatment, but decreased at 24 h in vivo. Furthermore, in vitro studies examining the effects of PGF and H_2_O_2_ on SOD expression in LECs, showed a similar pattern with increased SOD at 2 h followed by an inhibition at 24 h. These results suggest that SOD is temporally regulated by PGF and ROS during luteolysis in cattle. The dose of H_2_O_2_ used in the present study to examine SOD1 protein and total SOD activity did not affect significantly the viability of LECs.

Previous in vivo studies in cows demonstrated that injection of a luteolytic dose of PGF induces a transient (1–2 h) increase in the partial pressure of oxygen (pO_2_) in the ovarian venous blood [11]. In addition, it has been shown that a functional PGF receptor (FPr) is present in bovine LECs, and that PGF acutely increased ROS production in these cells [6]. Moreover, the rat CL produces significant amounts of ROS [24] and increases ROS (H_2_O_2_) generating capacity within a few hours after injection of a luteolytic dose of PGF [8,9]. In the present study, PGF stimulated the expressions of *SOD1* mRNA, protein, and total SOD activity at 2 h in bovine CL tissue and LECs. The temporal pattern of SOD expression suggests a role for ROS and SOD during the beginning functional luteolysis in cows. On the other hand, SOD acts as an antioxidant enzyme, and together with catalase, protects the endothelium of a variety of tissues against ROS [25]. Taken together, SOD and ROS seem to be involved in the luteolytic cascade induced by PGF in bovine CL.

ROS have been implicated in luteolysis due to their ability to increase uterine PGF [10], to decrease P_4_ biosynthesis [15] and to induce apoptosis in LSCs [26]. The intraluteal production of ROS is regulated by SOD [15]. SOD catalyzes the dismutation of superoxide into oxygen and H_2_O_2_[12]. SOD1 inhibition increases the steady-state levels of superoxide [27]. On the other hand, the presence of superoxide anions can induce intracellular SOD1. In the present study, H_2_O_2_ stimulated the expressions and activity of SOD1 at 2 h in bovine LECs. Sander et al. demonstrated an increase in total SOD activity and a decrease in catalase activity during CL regression in mice [28]. Since H_2_O_2_ has the capacity to increase PGF production in bovine LSCs [29] and LECs (the present study), as well as to induce apoptosis in bovine LSCs and human umbilical vein endothelial cells [29,30], increased levels of H_2_O_2_ may be crucial for luteal regression. The above findings also suggest that a local increase of ROS within the bovine CL facilitates the local production of PGF. Moreover, the concomitant increase of SOD and ROS may be due to the strong stimulatory effect of ROS on SOD in response to the increasing levels of PGF within the CL.

Although luteolytic PGF is derived from the uterus in many species, including ewes [31] and cows [2], a considerable amount of PGF is also synthesized by the CL [32] and LECs represent an important source of PGF [18]. Previous studies have reported that PGF increases the production of ROS in rats [9,24] and cow in vivo [11]. Interestingly, ROS has been demonstrated to stimulate PGF production in the CL of rats [33], cows [29] and human [10]. In the present study, H_2_O_2_ stimulated PGF production in cultured bovine LECs. The above results suggest the presence of a positive feedback loop between PGF and ROS in the bovine CL during luteolysis. Also, the increase of intraluteal PGF induced by ROS seems to be crucial for promotion of luteal regression in cow.

The present study demonstrates that PGF and H_2_O_2_ inhibited the expression of SOD1 protein in bovine LECs cultured for 24 h. The inhibitory effects of PGF and H_2_O_2_ on SOD expression at 24 h contrasted with their stimulatory effects at 2 h. The increase in SOD observed at 2 h seems to be the result of an acute stimulatory effect of PGF on ROS production by LECs. However, the mechanism by which PGF and H_2_O_2_ inhibit the expression of SOD1 at 24 h of incubation is unclear. SOD catalyzes the dismutation of superoxide into H_2_O_2_ and oxygen, to maintain low-state levels of superoxide [12]. Therefore, a reduction of SOD by PGF and H_2_O_2_ at 24 h may enhance intraluteal ROS or superoxide radical accumulation for the promotion of structural luteolysis [29,30]. In support of such an idea, an accumulation of superoxide radicals and a decrease in SOD levels are associated with the inhibition of luteal P_4_ secretion [7] and apoptotic cell death [34].

Furthermore, SOD1 protein was localized in LECs (Additional file 1: Figure S1) and other types of luteal cells (Additional file 2: Figure S2). A robust SOD1 protein expression was detected in bovine CL tissue after PGF treatment (Additional file 2: Figure S2). These findings suggest that not only SOD1 in LECs but also in other types of luteal cells including LSCs are regulated during PGF induced luteolysis. Further studies are needed to clarify the relative contribution of LSCs in total luteal SOD during luteolysis in cow.

## Conclusions

SOD is regulated by PGF and ROS in bovine LECs. SOD may play roles in controlling intraluteal PGF and ROS action during functional and structural luteolysis in cows.

## Competing interests

The authors declare that they have no competing interests.

## Authors’ contributions

HVV participated in the in vitro experiments and drafted the manuscript. TJA conceived of the study, participated in its design and performed in vivo and in vitro experiments. He also helped to draft the manuscript. ShY participated in data analysis and drafted the manuscript. HA carried out the in vitro experiments. KO participated in experimental design and discussion. All authors read and approved the final manuscript.

## Supplementary Material

Additional file 1 **Figure S1.** Immunohistochemical examination of SOD1 in bovine luteal tissue. For localization of SOD1, bovine corpus luteum tissues was fixed with 10% phosphate buffer formalin, embedded with paraffin and cut into 4 micrometers of thickness. For antigen retrieval, sections were incubated in Tris-EDTA buffer (pH 9.0) for 15 min at 98^0^C. Normal horse serum blocking solution was used for inhibition of nonspecific bindings. The slides were then incubated with or without (negative control) SOD1 antibody raised in goat (Santa Cruz; sc-8637) at a dilution rate of 1/200. After washing, sections were incubated with biotinylated rabit anti goat IgG serum as the second antibody (1/4000). Horseradish peroxidase (HRP)-conjugated ABC (Vector Laboratory Inc., Burlingame, CA, USA) was applied to the section at room temperature for 30 min. The binding sites were visualized using 0.02% 3,3^’^- diaminobenzidine tetrahydrochloride (DAB) in 50 mM Tris–HCl (pH 7.4) containing 0.02% H_2_O_2_. After immunohistochemical staining, the sections were lightly counterstained with Mayer’s hematoxylin. The sections were washed in distilled water, dehydrated in a graded series of ethanol, and cleared in xylene, coverslipped and observed under light field microscope. Immunohistochemical representative pictures of SOD1 were shown. Picture A showed the negative control (magnification: 200x). Picture B (magnification: 200x) and C (magnification: 400x) showed the localization of SOD1 (brown color) in the cytoplasm of luteal endothelial cells. Red arrows indicated cells with strong signal while black arrows indicated cells with weak signal. Click here for file

Additional file 2 **Figure S2.** Immunohistochemistry of SOD1 in bovine luteal tissue after PGF injection. The method for detection of SOD1 in bovine CL tissue after PGF injection was similar to that described above in the “Additional file 1: Figure S1” section. Immunohistochemical representative pictures of SOD1 were shown. Picture A was positive staining while picture B was negative control. SOD1 protein expression (brown color) in cytoplasm of luteal cells was robust after PGF-injection. Bar=50 μm. Click here for file
