# Correction: Roles of prostaglandin F2alpha and hydrogen peroxide in the regulation of Copper/Zinc superoxide dismutase in bovine corpus luteum and luteal endothelial cells

**DOI:** 10.1186/1477-7827-11-82

**Published:** 2013-09-06

**Authors:** Hai V Vu, Seunghyung Lee, Tomas J Acosta, Shin Yoshioka, Hironori Abe, Kiyoshi Okuda

**Affiliations:** 1 Laboratory of Reproductive Physiology, Graduate School of Natural Science and Technology, Okayama University, Okayama 700-8530, Japan

## Correction

After publication of this work [1], we noted that we inadvertently failed to include the complete list of all coauthors. The full list of authors has now been added and the Authors’ contributions, Competing interests and Acknowledgment section have been modified accordingly.

## Competing interests

The authors declare that they have no competing interests.

## Authors’ contributions

HVV participated in the *in vitro* experiments, data analysis and drafted the manuscript. SL carried out the *in vitro* experiments, data analysis and drafted the manuscript. ShY participated in data analysis and drafted the manuscript. HA carried out the *in vitro* experiments. KO participated in experimental design and discussion. TJA conceived of the study, participated in its design and performed *in vivo* and *in vitro* experiments. He also helped to draft the manuscript. All authors read and approved the final manuscript.
