# Supplementary figures and images for: Roles of prostaglandin F2alpha and hydrogen peroxide in the regulation of Copper/Zinc superoxide dismutase in bovine corpus luteum and luteal endothelial cells
Source: Reprod Biol Endocrinol. 2012 Oct 26;10:87. doi: 10.1186/1477-7827-10-87 (PMC3545964; doi:10.1186/1477-7827-10-87)

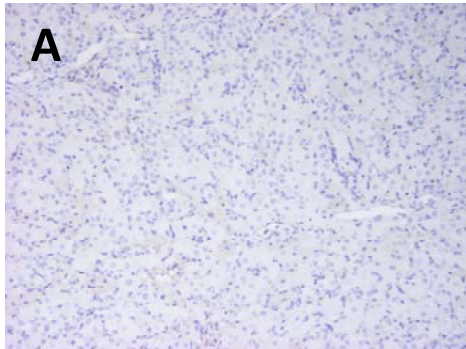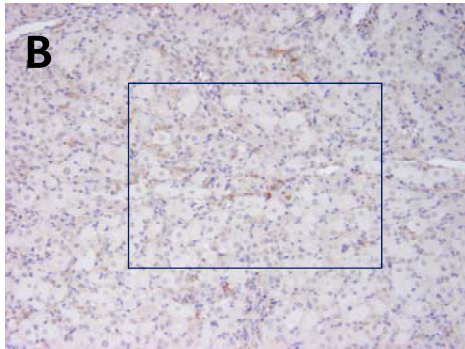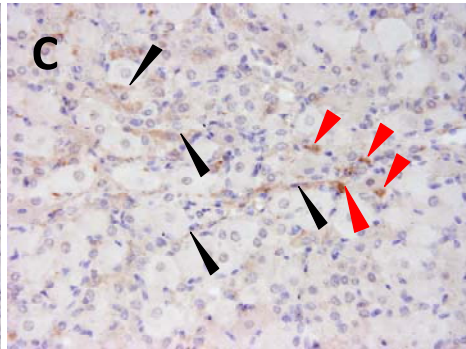

Supplement: Additional file 1 — Figure S1. Immunohistochemical examination of SOD1 in bovine luteal tissue. For localization of SOD1, bovine corpus luteum tissues was fixed with 10% phosphate buffer formalin, embedded with paraffin and cut into 4 micrometers of thickness. For antigen retrieval, sections were incubated in Tris-EDTA buffer (pH 9.0) for 15 min at 980C. Normal horse serum blocking solution was used for inhibition of nonspecific bindings. The slides were then incubated with or without (negative control) SOD1 antibody raised in goat (Santa Cruz; sc-8637) at a dilution rate of 1/200. After washing, sections were incubated with biotinylated rabit anti goat IgG serum as the second antibody (1/4000). Horseradish peroxidase (HRP)-conjugated ABC (Vector Laboratory Inc., Burlingame, CA, USA) was applied to the section at room temperature for 30 min. The binding sites were visualized using 0.02% 3,3’- diaminobenzidine tetrahydrochloride (DAB) in 50 mM Tris–HCl (pH 7.4) containing 0.02% H2O2. After immunohistochemical staining, the sections were lightly counterstained with Mayer’s hematoxylin. The sections were washed in distilled water, dehydrated in a graded series of ethanol, and cleared in xylene, coverslipped and observed under light field microscope. Immunohistochemical representative pictures of SOD1 were shown. Picture A showed the negative control (magnification: 200x). Picture B (magnification: 200x) and C (magnification: 400x) showed the localization of SOD1 (brown color) in the cytoplasm of luteal endothelial cells. Red arrows indicated cells with strong signal while black arrows indicated cells with weak signal. [file 1477-7827-10-87-S1.pdf]

**A**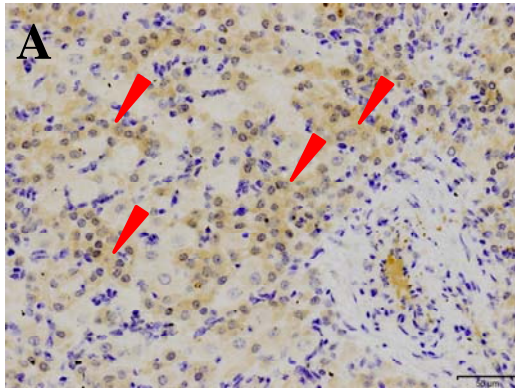**B**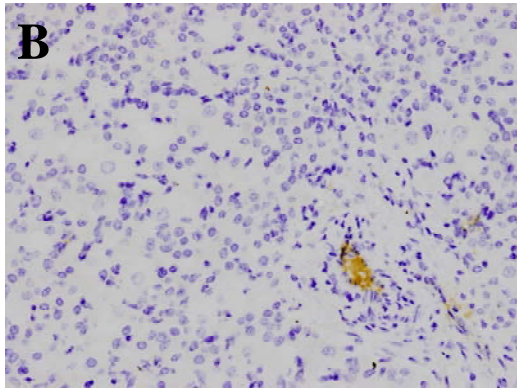

Supplement: Additional file 2 — Figure S2. Immunohistochemistry of SOD1 in bovine luteal tissue after PGF injection. The method for detection of SOD1 in bovine CL tissue after PGF injection was similar to that described above in the “Additional file 1: Figure S1” section. Immunohistochemical representative pictures of SOD1 were shown. Picture A was positive staining while picture B was negative control. SOD1 protein expression (brown color) in cytoplasm of luteal cells was robust after PGF-injection. Bar=50 μm. [file 1477-7827-10-87-S2.pdf]
